# Mortality among blood donors seropositive and seronegative for Chagas disease (1996–2000) in São Paulo, Brazil: A death certificate linkage study

**DOI:** 10.1371/journal.pntd.0005542

**Published:** 2017-05-18

**Authors:** Ligia Capuani, Ana Luiza Bierrenbach, Airlane Pereira Alencar, Alfredo Mendrone, João Eduardo Ferreira, Brian Custer, Antonio Luiz P. Ribeiro, Ester Cerdeira Sabino

**Affiliations:** 1 Department of Infectious Diseases, School of Medicine—University of Sao Paulo—FMUSP, Sao Paulo, Sao Paulo, Brazil; 2 Institute of Education and Research, Hospital Sirio Libanes, Sao Paulo, Sao Paulo, Brazil; 3 Department of Statistics, Institute of Mathematics and Statistics, University of Sao Paulo—IME-USP, Sao Paulo, Sao Paulo, Brazil; 4 Fundação Pró-Sangue—Hemocenter of Sao Paulo, Sao Paulo, Sao Paulo, Brazil; 5 Department of Computer Science, Institute of Mathematics and Statistics, University of Sao Paulo—IME-USP, Sao Paulo, Sao Paulo, Brazil; 6 Epidemiology and Health Policy Research, Blood Systems Research Institute—BSRI, San Francisco, California, United States of America; 7 Hospital das Clínicas and School of Medicine, Universidade Federal de Minas Gerais—UFMG, Belo Horizonte, Minas Gerais, Brazil; 8 Department of Infectious Diseases, School of Medicine and Tropical Medicine Institute—University of Sao Paulo—FMUSP, Sao Paulo, Sao Paulo, Brazil; Imperial College London, UNITED KINGDOM

## Abstract

**Background:**

Individuals in the indeterminate phase of Chagas disease are considered to have mortality rates similar to those of the overall population. This study compares mortality rates among blood donors seropositive for Chagas disease and negative controls in the city of São Paulo, Brazil.

**Methodology/principal findings:**

This is a retrospective cohort study of blood donors from 1996 to 2000: 2842 seropositive and 5684 seronegative for Chagas disease. Death status was ascertained by performing probabilistic record linkage (RL) with the Brazil national mortality information system (SIM). RL was assessed in a previous validation study. Cox Regression was used to derive hazard ratios (HR), adjusting for confounders. RL identified 159 deaths among the 2842 seropositive blood donors (5.6%) and 103 deaths among the 5684 seronegative (1.8%). Out of the 159 deaths among seropositive donors, 26 had the 10th International Statistical Classification of Diseases and Related Health Problems (ICD-10) indicating Chagas disease as the underlying cause of death (B57.0/B57.5), 23 had ICD-10 codes (I42.0/I42.2/I47.0/I47.2/I49.0/I50.0/I50.1/ I50.9/I51.7) indicating cardiac abnormalities possibly related to Chagas disease listed as an underlying or associated cause of death, with the others having no mention of Chagas disease in part I of the death certificate. Donors seropositive for Chagas disease had a 2.3 times higher risk of death due to all causes (95% Confidence Interval (95% CI), 1.8–3.0) than seronegative donors. When considering deaths due to Chagas disease or those that had underlying causes of cardiac abnormalities related to Chagas disease, seropositive donors had a risk of death 17.9 (95% CI, 6.3–50.8) times greater than seronegative donors.

**Conclusions/significance:**

There is an excess risk of death in donors seropositive blood for Chagas disease compared to seronegative donors. Chagas disease is an under-reported cause of death in the Brazilian mortality database.

## Author summary

Chagas disease is classified as one of the 17 most important neglected diseases by the World Health Organization (WHO). The majority of infected individuals live in 21 countries of Central and South America. Estimates of mortality attributable to Chagas disease vary considerably (between 0.2% and 19.2% annually). To improve the measurement of disease burden and policy decision-making in regard to treatment availability, more accurate estimates of mortality, especially among otherwise asymptomatic seropositive individuals are needed,. This study compares mortality rates and causes of death of asymptomatic blood donors who tested seropositive for Chagas disease and seronegative for all screening tests conducted in Brazil. Mortality status was ascertained by linking donor names with the Brazilian national mortality information system (SIM). The study found that donors who tested Chagas disease seropositive had risk of death from all causes 2.3 (95% CI, 1.8–3.0) times greater than seronegative ones. The data also suggest that the SIM may underestimate the total number of deaths attributable to Chagas disease in Brazil.

## Introduction

Chagas disease was first described in 1909 by the Brazilian scientist Carlos Ribeiro Justiniano Chagas (1879–1934). The etiologic agent that causes Chagas disease is the protozoan parasite *Trypanosoma cruzi* (*T*. *cruzi*). The parasite is transmitted by the reduviid bug, a member of the Triatomine subfamily, popularly known as the “kissing bug”. Transmission may also occur by blood or organ transfusion, contaminated food, from mother to child and due to laboratory accidents [1]. Classified as one of the 17 most important neglected diseases by the World Health Organization (WHO), estimates of the number of infected individuals in the world have decreased from approximately 20 million in 1981 [1], to 7–8 million in 2014 [2].

In Brazil, from 1980 to 2000 there was a great effort by health authorities to eradicate the insect and to stop transfusion transmission. The reduction in disease burden is predominantly a result of improved vector control. The main measures of disease control implemented were the elimination of *Triatoma infestans* peri and in-house habitat by the use of insecticides, housing stock improvement, sanitary education, and the adoption of screening tests among blood donors to detect the parasite and thus avoid transmission [3]. Donor screening for *T cruzi* is mandatory in all Latin American countries with more than 92% of screening coverage of the blood supply.[4] The prevalence of *T cruzi* has declined among first time blood donors in Brazil, from 0.52% in 1996 [5] to 0.14% in 2008 [6].

In humans, Chagas disease manifests in two phases, acute and chronic. The acute phase has mild symptoms that may last for approximately two months. In the chronic phase, the majority of cases are of the asymptomatic indeterminate form, which may last a lifetime. Individuals in the chronic phase of the disease with normal electrocardiogram (EKG) and normal chest and esophagus X-rays are considered individuals in the indeterminate form of the disease [7]. Whereas, clinically apparent chronic disease form is divided into a cardiac, gastrointestinal, or combined form [8]. The most important health consequence of Chagas disease is cardiomyopathy, which over a lifetime occurs in 20–40% of infected persons with an incidence rate of 1.85% per person-year [9]. In Brazil, it is estimated that in 2013 there were 2 to 3 million infected individuals [10], of which 600,000 had cardiac and/or gastrointestinal complications. The disease is also responsible for approximately 6,000 deaths per year in Brazil, i.e. 0.6% of all deaths that occur in the country [11]. This estimate is based on analyses of data from the National Mortality Information System (SIM) from 1999 to 2007, in which records with 10th International Statistical Classification of Diseases (ICD-10) codes B57 and all subcategories (B57.0 to B57.5) marked on the underlying and/or associated causes of death field were selected [11, 12].

One of the critical challenges for Chagas disease control is that many individuals with the indeterminate form of Chagas disease are not aware that they are infected, so that these individuals are potential onward *T*. *cruzi* transmitters and may even die without being diagnosed with the disease. Mortality-associated with Chagas disease is still inadequately estimated, particularly for those with the indeterminate form of the disease. Important unanswered questions include: do persons with indeterminate disease have an increased risk of dying? Studies that compare mortality among seropositive and seronegative individuals can help reduce this knowledge gap as described in a systematic review and meta-analysis published in 2016 [13]. The objective of the study was to compare death rates during an up to 14 year follow-up period among Chagas disease seropositive blood donors and seronegative blood donors identified in the period 1996–2000 in the city of São Paulo, Brazil.

## Methods

This is a retrospective cohort study. This study compared mortality rates and causes of death in blood donors at Fundação Pró-Sangue—Hemocentro de São Paulo (FPS), one of the largest blood banks in State of São Paulo, located in São Paulo city.

### Subjects

FPS is a reference center in Latin America, and the proportion of the supply collected is approximately 33% of all donated blood used in the metropolitan region of São Paulo city. FPS is a reference center for the Pan-American Health Organization (PAHO),World Health Organization (WHO) and the Brazilian Ministry of Health, it collects and processes 90,000 blood units annually that are used by more than 100 health institutions [14].

Blood donors constitute a particular population in which, if infected, seropositive individuals are mostly asymptomatic and therefore are more likely to be in the indeterminate form Chagas disease. Although donation eligibility assessment does not include EKG or X rays, blood donors go through an interview before donating and requirements to donate include being in good health with no evidence of or reported history of Chagas disease or cardiomyopathies.

During the study period (1996–2000) FPS screened all donors with 3 serological assays (indirect immunofluorescence (IIF), indirect hemaglutination (IHA), and enzyme-linked immunosorbent assay (ELISA)) [15]. Donors reactive on all 3 assays were considered confirmed positive. Using blood bank computerized records, all confirmed seropositive donors for Chagas disease were included (n = 2,842) and a random sample of blood donors seronegative for all screening tests performed at FPS during the same time period was selected, that is, 2 seronegative donors to each seropositive matched by year of donation (n = 5,684). During the study period, FPS routinely performed the following serology screening tests on all donations before releasing units for transfusion: syphilis, Human immunodeficiency virus (HIV), Human T-cell lymphotropic viruses I and II (HTLV), Hepatitis C Virus (HCV), Hepatitis B Virus (HBV), and Chagas disease.

### Ascertainment of death status

In order to ascertain the death status of the study population, a record linkage (RL) was performed to link records of the FPS database with the Brazil national mortality information system (Sistema de Informações sobre Mortalidade—SIM) records. As Brazil does not have a unique national identity number, a probabilistic RL was performed using open source software. Patients’ name, mother´s name and date of birth were the chosen linking variables. One of the advantages of the use of this software in our context is that it links names and other variables through the use of a Soundex system which is specific for Portuguese phonetics (Reclink III, version 3.0.4 4005, Rio de Janeiro, Brazil) [16].

The RL procedure used here has been described in depth in a previous publication [17]. In this previous study, we assessed the RL methodology validity by assembling a list of blood donors with known vital status. Sensitivity and specificity of the RL method to detect mortality were 94% (95%CI, 90–97%) and 100% (95%CI, 98%-100%), respectively.

In the SIM database, records containing the variables patients’ name, mother´s name, date of birth and address of residence were only available for the years 2001 to 2009 (SIM version extracted on 11/01/2010, Brasilia, Brazil). FPS donors included in the study donated blood during the period of 1996 to 2000, this means that even though we were able to retrospectively follow all donors for at least nine years, up to first five years of follow-up were not available in SIM. For the cohort that donated blood in 1996, we missed the first five years, for the 1997 cohort, we missed the first four years and so forth. In other words, irrespective of their Chagas serology status, we do not know whether those who donated blood in 1996, for example, died in the period of 1996 to 2000. However, based on findings of seropositive and seronegative individuals who donated blood in 2000 and for whom we only missed deaths that might have happened during the year of their blood donation, we found that deaths were extremely rare in the first year after donation. Because of this finding and with a view of circumscribing this data limitation, we made the analytical assumption that nobody in our cohort died in the years 1996 to 2000.

### Description of causes of death

For the descriptive analysis, the underlying causes of death of seropositive and seronegative donors were grouped by chapters in the ICD-10.

For the multivariate analysis, causes of death were analyzed according to three groups: A) Confirmed deaths due to Chagas disease, B) Confirmed and possible deaths due to Chagas disease, and C) Death due to all causes. Group A is hierarchically a subgroup of group B, which is a subgroup of group C. “Confirmed deaths due to Chagas disease” refers to deaths recorded with ICD-10 codes B57.0—B57.5 listed on the underlying or the associated causes of death fields. “Confirmed and possible deaths due to Chagas disease” refer to deaths recorded with ICD-10 codes B57.0—B57.5 plus other codes indicating cardiac abnormalities that are possibly due to Chagas disease listed on the underlying or the associated causes of death fields: I42.0—Dilated cardiomyopathy, I42.2—Other hypertrophic cardiomyopathy, I47.0—Re-entry ventricular arrhythmia, I47.2—Ventricular tachycardia, I49.0—Ventricular fibrillation and flutter, I50.0—Congestive heart failure, I50.1—Left ventricular failure, I50.9—Heart failure, unspecified and I51.7—Cardiomegaly.

### Statistical analysis

Comparisons of characteristics of donors that were seropositive and seronegative or that were alive or dead at the end of the follow-up period were performed using two-tailed Fisher Exact tests or Chi-squared tests, as appropriate. Mortality rates were calculated by dividing the number of donors that died (numerator) by the total person-years at risk of dying during the follow-up period, i.e. from donation to death or to the end of the study period (denominator). Survival of seropositive and seronegative donors over time was graphically assessed by Kaplan-Meier (KM) curves and statistical differences tested with the Logrank test.

Age and sex were the only two demographic variables available for analysis in the FPS database that represented potential confounders of the association between the outcomes variables and the main explanatory variable under investigation, i.e. serological status. Age was analyzed a categorical variable (18–29 years, 30–39 years, 40–49 years and >50 years).

Multiple Cox Regression models were used to derive hazard ratios (HR) for the outcomes studied, adjusting for confounders and testing for interactions, where appropriate. Two sets of analyses were performed one for the whole cohort and another excluding Chagas disease positive donors co-infected with one or more of the following infections: syphilis, HIV, HTLV, HCV and HBV.

Regression models containing all potential predictors were estimated. The predictor variables available for the regression models were age group and sex. Stepwise backward selection procedures were used to remove variables. Variables were removed with Wald test p-values ≥0.2. The remaining variables were then successively removed based on lack of evidence of confounding and their contribution to the models. Variables whose removal from the models caused substantial changes (defined as >10%) in the HRs were retained, as were variables whose removal incurred significantly reduced model fit based on likelihood ratio tests (p-values <0.05). For each outcome and potential risk factor, the proportional hazards assumption was tested statistically using Schoenfeld residuals and by examining for parallel curves in log-log plots. Schoenfeld residuals tests were not statistically significant and the curves were mostly parallel, indicating no evidence of violation of the proportional hazards assumption. In addition, the proportional hazard was assessed by including a time-dependent interaction term in the multivariable Cox models and by testing the statistical significance of this interaction using the likelihood ratio test. Consistently, results indicated no evidence of violation of the assumption. Statistical analyses were conducted using Stata version 13.1 (StataCorp LP, College Station, TX, USA) software [18].

### Ethical approval

Ethical approval for this study was obtained from the Ethics Research Committee of Clinical Hospital of the School of Medicine—University of Sao Paulo, HC-FMUSP (CAAE:03882512.4.0000.0065). HC-FMUSP Ethics Research Committee determined it unnecessary to obtain informed consent from each individual for reviewing medical records, given the difficulty of obtaining such consent and because the study was conducted using 2 existing databases. The identifiers were removed from database after the probabilistic RL was performed.

## Results

### Probabilistic record linkage results

Out of 1,354,569 blood donors to FPS during the study time period, 2,842 confirmed positive cases and 5,684 controls (matched by year of donation) were assessed for death status and are described on Table 1.

**Table 1:** Comparison of blood donors seronegative and seropositive for Chagas disease, at FPS, São Paulo, Brazil, from 1996–2000, by age, gender, year of blood donation and mortality.

	All subjects	Seropositive	Seronegative	p-value
	N = 8,526	N = 2,842	N = 5,684	
	n (%)	n (%)	n (%)	
Gender				0.114
Male	6,022 (70.6)	1,976 (69.5)	4,046 (71.2)	
Female	2,504 (29.4)	866 (30.5)	1,638 (28.8)	
Age groups (years)				<0.001
18–29	3,523 (41.4)	646 (22.7)	2,886 (50.8)	
30–39	2,424 (28.4)	795 (28.0)	1,629 (28.7)	
40–49	1,726 (20.3)	893 (31.4)	833 (14.6)	
>50	844 (9.9)	508 (17.9)	336 (5.9)	
Year of donation				1,000
1996	2,310 (27.1)	771 (27.1)	1,539 (27.1)	
1997	2,153 (25.3)	717 (25.2)	1,436 (25.3)	
1998	1,735 (20.3)	578 (20.4)	1,157 (20.3)	
1999	1,215 (14.2)	406 (14.3)	809 (14.2)	
2000	1,113 (13.1)	370 (13.0)	743 (13.1)	
Mortality	262 (3.1)	159 (5.6)	103 (1.8)	<0.001
Average age at death	48.6 ± 11.6	50.8 ± 11.3	45.1 ± 11.3	

The RL method identified a total of 262 deaths, 159 (5.6%) among the seropositive blood donors and 103 (1.8%) among the seronegative. Mortality was significantly higher in seropositive donors.

Out of the 2842 Chagas disease seropositive donors, 128 (4.5%) were also seropositive for at least one of the other tested infections. Syphilis (51 donors) was the most common co-infection, followed by HBV (35 donors) and HCV (26 donors), HTLV (21 donors) and HIV (2 donors). A total of 12 donors were co-infected with Chagas disease plus two of the other infections.

### Causes of death

The distribution of the underlying causes of death of seropositive and seronegative donors, as classified by ICD-10 chapters, is shown in Table 2. Distribution by subcategories of ICD-10 is available in a supplemental table (S1 Table). Seropositive donors died more frequently of diseases classified with codes of Chapter IX—Diseases of circulatory system and Chapter I—Certain infectious and parasitic diseases as compared to seronegative donors, On the other hand, seronegative donors died more frequently of external causes (Chapter XX).

**Table 2:** Comparison of deceased blood donors seronegative and seropositive for Chagas disease, at FPS, São Paulo, Brazil, from 1996–2000, by ICD-10 chapters of the underlying causes of death.

Basic Cause of Death Description	Seroposite	Seronegative	p-value
	n = 159	n = 103	p<0.001
Chapter I—Certain infectious and parasitic diseases	32	3	
Chapter II—Neoplasms	22	17	
Chapter IV—Endocrine, nutritional and metabolic diseases	0	2	
Chapter V—Mental and behavioural disorders	1	0	
Chapter VI—Diseases of the nervous system	0	1	
Chapter IX—Diseases of the circulatory system	58	20	
Chapter X—Diseases of the respiratory system	9	2	
Chapter XI—Diseases of the digestive system	8	8	
Chapter XIV—Diseases of the genitourinary system	2	0	
Chapter XVII—Congenital malformations, deformations and chromosomal abnormalities	0	1	
Chapter XVIII—Symptoms, signs and abnormal clinical and laboratory findings, not elsewhere classified	9	8	
Chapter XX—External causes of morbidity and mortality	18	41	

Out of the 262 donors that died, 26 had codes B57 listed as the underlying cause of death and were therefore classified as "Confirmed deaths due to Chagas disease". All of them were seropositive for Chagas disease. There was no record with B57 listed as an associated cause of death, but there was one seropositive donor with code B57 listed on part II (co-morbidities) on the death certificate.

A total of 49 donors were classified as "Confirmed and possible deaths due to Chagas disease". This number includes all 26 donors classified as "Confirmed deaths due to Chagas disease" plus 23 donors that had codes indicating cardiac abnormalities possibly related to Chagas disease, listed as an underlying or associated cause of death. Codes indicating cardiac abnormalities possibly related to Chagas disease were listed as associated causes of death in 16 (61.5%) of the 26 donors classified as "Confirmed deaths due to Chagas disease". Out of the other 236 donors that were not classified as "Confirmed deaths due to Chagas disease", only 23 (9.8%) had any of these codes listed as an underlying or associated cause of death (p<0.001). Moreover, these codes were listed in 35 (22%) of the 159 deaths of seropositive donors and in only 4 (3.9%) of the 103 deaths of seronegative donors (p<0.001).

The underlying causes of death of the 49 donors classified as "Confirmed and possible deaths due to Chagas disease" is shown in Table 3. It was unexpected to find code B56.9 (African trypanosomiasis) in a donor seropositive for *T*. *cruzi*. While possible, it may also have been a coding mistake.

**Table 3:** Underlying causes of death of all donors included in the study classified as “Confirmed and possible deaths due to Chagas disease”.

Underlying causes of death	Seropositive	Seronegative	Total	
			N = 49	
			N	(%)
**Confirmed deaths due to Chagas disease**				
B57.2—Chagas disease (chronic) with heart involvement	22	0	22	(44.90)
B57.3—Chagas disease (chronic) with digestive system involvement	3	0	3	(6.12)
B57.4—Chagas disease (chronic) with nervous system involvement	1	0	1	(2.04)
**Cardiac abnormalities possibly related to Chagas**				
I42.0—Dilated cardiomyopathy	5	1	6	(12.24)
I50.0—Congestive heart failure	1	0	1	(2.04)
I51.7—Cardiomegaly	3	0	3	(6.12)
**Other cardiac/circulatory causes**				
I11.0—Hypertensive heart disease with (congestive) heart failure	1	1	2	(4.08)
I21.9—Acute myocardial infarction, unspecified	2	0	2	(4.08)
I24.8—Other forms of acute ischaemic heart disease	1	0	1	(2.04)
I25.1—Atherosclerotic heart disease	1	0	1	(2.04)
I25.9—Chronic ischaemic heart disease, unspecified	1	0	1	(2.04)
I51.4—Myocarditis, unspecified	1	0	1	(2.04)
I71.8—Aortic aneurysm of unspecified site, ruptured	1	0	1	(2.04)
**Other causes**				
B56.9—African trypanosomiasis, unspecified	1	0	1	(2.04)
C44.9—Malignant neoplasm: Malignant neoplasm of skin, unspecified	1	0	1	(2.04)
C50.9—Malignant neoplasm: Breast, unspecified	1	0	1	(2.04)
F10.2—Mental and behavioural disorders due to use of alcohol: dependence syndrome	1	0	1	(2.04)

### Survival analysis

**Fig 1:**
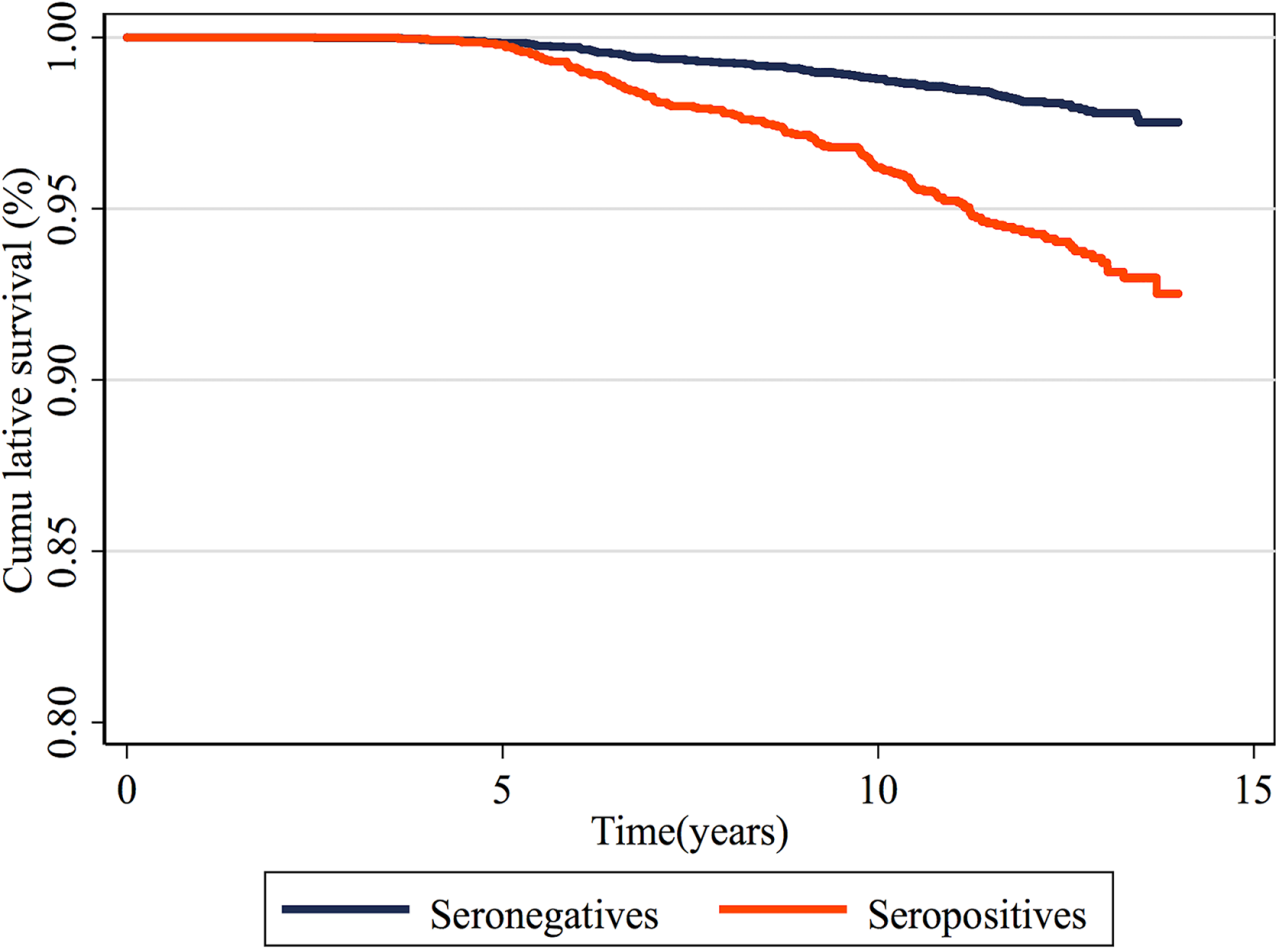
Cumulative survival of blood donors at FPS, São Paulo, Brazil, from 1996–2000, by *T*. *cruzi* serology status.

**Fig 2:**
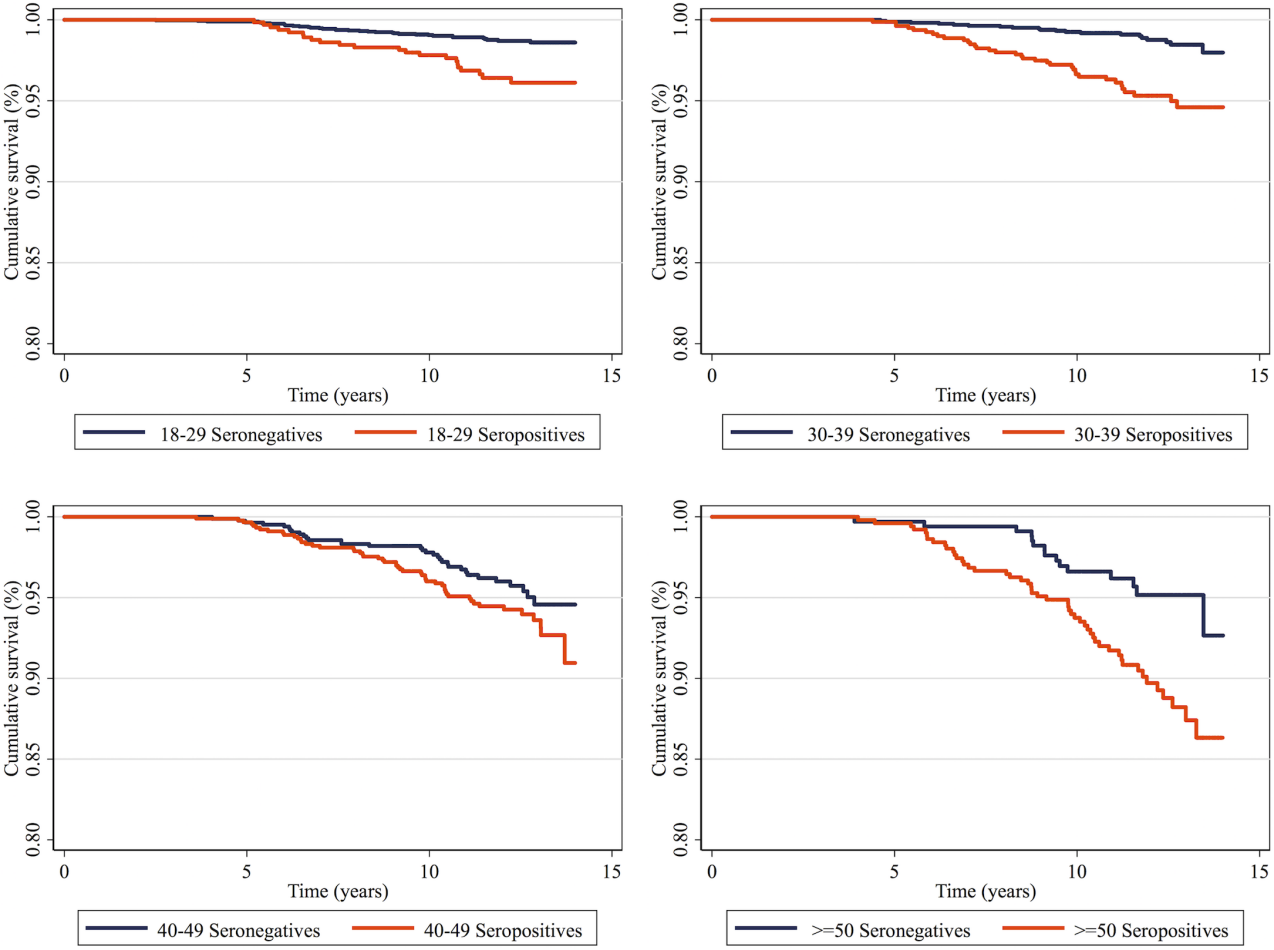
Cumulative survival of blood donors at FPS, São Paulo, Brazil, from 1996–2000, by *T*. *cruzi* serology status and age group.

Donors’ cumulative survival according to serology, without excluding co-infected individuals, is shown in Fig 1 and according to serology and age group in Fig 2. Survival curves of seronegative donors were better than those of seropositive ones (p<0.001). The same pattern was observed for the KM curves by age group. For the age group 40–49 years the difference did not reach statistical significance (p = 0.097).

### Multivariate analysis

In the multivariate analysis, estimated crude and adjusted hazard ratios were calculated for each of the three different case definitions: A) Confirmed deaths due to Chagas disease B) Confirmed and possible deaths due to Chagas disease and C) Death due to all causes (Table 4). As there were no confirmed deaths due to Chagas disease in the seronegative group, HRs could not be estimated for this case definition. The age adjusted HR for mortality in seropositive donors was 2.3 (95%CI 1.8–3.0) times higher than seronegative ones when deaths due to all causes were considered and the age and sex adjusted HR for mortality was 17.9 (95%CI 6.3–50.8) times higher for confirmed and possible deaths due to Chagas disease.

**Table 4:** Mortality numbers and rates for the cohorts of individuals seropositive for Chagas disease or seronegative for all screening tests at FPS, São Paulo, Brazil, from 1996–2000. Estimated crude and adjusted hazard ratios, for three different mortality case definitions.

Case definitions	Number of deaths		Mortality rates		Crude HR	Adjusted HR***
			100,000 PY**		(95%CI)	(95%CI)
	Sero+*	Sero-*	Sero+*	Sero -*	p value	p value
	N = 2,842	N = 5,684	PY = 33,295.042	PY = 67,172.69		
**A. Confirmed deaths due to Chagas disease**						
ICD10 codes B57	26	0	78.1	0	-	-
(Underlying or associate causes of death)						
**B. Confirmed and possible deaths due to Chagas disease**						
ICD10 codes B57 I420 I422 I470 I472 I490 I500 I501 I517 I509	45	4	135.2	6	22.8 (8.2–63.4)	17.9 (6.3–50.8)
(Underlying or associate causes of death)					<0.001	<0.001
**C. Death due to all causes**	159	103	477.4	153.4	3.1 (2.4–4.0)	2.3 (1.8–3.0)
** **					<0.001	<0.001

* Sero+ = Seropositive donors for Chagas disease screening tests at FPS—Sero- = Seronegative donors for all screening tests at FPS

** PY–persons-year

** Adjustments: Model B was adjusted for age group and gender; Model C was adjusted for age group.

Excluding co-infected donors did not appreciably change the results. When multivariate analyses were performed excluding the 128 co-infected donors, seropositive donors had a HR 2.2 (95%CI = 1.7–2.9, p-value<0.001) times higher than seronegative ones for all cause deaths and a HR 16.1 (95%CI = 5.6–45.9, p-value<0.001) times higher when considering confirmed and possible deaths due to Chagas disease.

## Discussion

In this study, we compared the mortality rates of blood donors identified as seropositive for Chagas disease and seronegative for all screening tests based on donation testing in the 5-year time period, 1996–2000. Our results show that seropositive blood donors had a risk of dying over the study time period 2.3 times greater than seronegative individuals. When considering only deaths due to Chagas disease or those that had underlying causes of cardiac abnormalities that could indicate Chagas disease, seropositive blood donors had a much higher risk of death than seronegative donors. In a recent systematic review, Cucunuba et al. analyzed 25 papers published on mortality of Chagas disease from 10,638 individuals and 53,346 persons-year of follow-up. Only six of those studies focused on asymptomatic patients [19–24], following a total of 4,393 individuals for 34,217 person-years of observation. Crude relative risk of mortality from these studies varied from 0.81 to 3.59.

In this study, we have been able to follow 8,526 individuals for 100,467 person-years of observation, doubling the data available and contributing to a more precise evaluation of the impact of Chagas disease-associated mortality among asymptomatic seropositive individuals.

Classifying mortality for persons who are initially identified as being in the indeterminate form is difficult because disease progression is slow, studies need large sample sizes and very long follow-up periods. Population studies are expensive, and in areas with low prevalence such as the city of São Paulo, with prevalence rates of less than 1% [5, 6], screening of a large number of individuals would be necessary to achieve a sufficient number of seropositive cases. We overcame this problem by focusing on blood donors that are routinely screened for Chagas disease and that data are available through computerized systems. In Brazil, the national mortality information system has a national coverage and the quality of its cause of death data has improved substantially over the last two decades [25–28].

A provocative finding is that cardiac causes of death with no mention of Chagas disease was significantly more common among seropositive donors. Indeed, of the 45 deaths due to cardiac disease among seropositive donors only 26 (58%) had Chagas disease listed on their death certificate as an underlying cause of death. This indicates that the impact of Chagas disease mortality in Brazil is underestimated when analyses of only death records that specifically mention Chagas disease as the underlying cause of death are conducted [11]. The fact that Chagas disease was not reported as an underlying or associated cause of death on the death certificate of 42% of seropositive donors that died due to cardiac causes demonstrates under ascertainment of Chagas disease pathogenesis, highlighting its status as a neglected disease. Given the context for donation test result notification in Brazil and because the seropositive donors were asymptomatic at time of donation, only those that returned to the blood bank to get their screening tests results would have been notified of being seropositive for *T*. *cruzi* infection.

The use of the probabilistic RL method implies we might have missed some deaths [17]. However, as reported previously by our group, the methodology used had a sensitivity of 94% and a specificity of 100%, making us confident that the deaths found do represent true matches. But the main limitation of our study was that death information recorded by name was not available in the mortality database for the years 1996 to 2000, so that we were unable to ascertain whether or not donors in our cohort died in the early years of follow-up. In the analysis, we could have removed donors from this period and analyzed only those donating from 2001 onwards, but we faced the problem that during the study years, donors seropositive for Chagas disease were progressively less frequent in our database [5]. This decrease is probably due to two reasons: 1) vector transmission control activities in the country causing a decreased overall number of seropositive individuals in the population [5], and 2) in order to increase blood donations, from 1995–2001 FPS conducted a campaign to recruit previous donors seronegative for all screening tests to donate again, so that the proportion of donors who had not donated before became increasingly smaller compared to repeated donors [29]. If we had used in our cohort blood donors only from 2001 onward, the number of seropositive cases would be substantially lower and the follow-up period would be shorter, with the potential to attenuate the results of the analyses of this slow progressing disease.

However, early deaths after donation were not frequent, as observed for those who donated in 1999 and 2000 for whom we were able to ascertain whether or not they had died after their first or second year of follow-up, respectively (see S2 Table). If we had had mortality data for the whole cohort period, we have no reason to suppose that there would be an excess of early deaths in seronegative donors that would counterbalance the excess of late deaths that we found in the seropositive ones. Moreover, because the proportion of ICD-10 chapter XX, external causes of death, were equivalent in the seropositive and seronegative groups, an additional level of assurance is provided that there was no clear differential bias in mortality ascertainment between these two groups. Even if the number of deaths has been underestimated by the RL method and available data in SIM, the impact would be expected to be non-differential indicating the findings of our study are robust. In summary, we do not believe that this limitation greatly affected our results.

Another limitation with greater potential impact on our results is the fact that data regarding socioeconomic status was not available in the blood bank database, so we could not control for this effect in our multivariate models. In general, persons with *T*. *cruzi* infection are mostly poor, less educated, and from rural areas. This combination of factors could lead to increase mortality for reasons such as less healthy lifestyles or access to care. In turn, this could lead to increased mortality. Yet, these individuals presented to blood donate and at the time were eligible and donated following the extensive health history assessment covering a range of topics, including current physical health, substance abuse history, and sexual history. Our analysis might have generated increased accuracy in the HR results if we had been able to adjust for socioecomic status.

Although our study is focused in the city of Sao Paulo, we believe it well represent Brazil. Internal migration was an important factor for population increase in the city of Sao Paulo [30]. Data regarding State of Birth is not available at FPS data system, nevertheless, in a previous study done by our group in the same center (Retrovirus Epidemiological Donor Study (REDS–II)), seropositive and seronegative donors were interviewed and individuals born in the State of Sao Paulo represented 13% of the seropositive and 55% of the seronegative blood donors [9].

We believe that most Chagas disease seropositive donors were in the indeterminate form of the disease. Individuals who present to donate blood have to report if they do not have any current serious conditions in a comprehensive questionnaire about their health status. Questions such as “Do you have a severe heart problem?” and “Do you have arrhythmia and use medication for this problem?” are part of the screening questionnaire. However, we do not know if any of the donors had abnormal EKGs or abnormalities identifiable by X-ray, in which case they would be classified in the clinical form of the chronic phase of the disease. The increased mortality findings have high relevance for common assumptions about Chagas disease morbidity. It is not uncommon to find articles in the literature that consider the indeterminate form of the disease as a benign disease not requiring close medical attention [20, 31, 32]. However, such studies are generally based on following a small number of people and often without appropriate comparison groups [13]. When clinicians read these articles they may feel justified in not treating their patients with the limited number of therapeutic options that are available. Because available pharmacotherapy is not very effective and all of them have potential important side effects [32, 33], clinicians may not want to treat indeterminate Chagas. Our findings suggest that this belief may be erroneous and that the indeterminate form, even in a relatively young population, is associated with higher mortality rates. Earlier treatment to reduce morbidity and mortality may be important. Chagas is a neglected disease and research is urgently needed in order to test new therapeutic options with fewer side effects and to find better correlates of disease progression.

In conclusion, this analysis shows that there is an excess risk of death in donors who test seropositive for Chagas disease compared to seronegative donors. Chagas disease is an under-estimated cause of death in the Brazilian National Mortality Information System.

## Supporting information

S1 Table Complete causes of death of blood donors at FPS, São Paulo, Brazil, from 1996–2000. (XLSX)

S2 Table Number of deaths by year of donation of blood donors at FPS, São Paulo, Brazil, from 1996–2000. (XLSX)

## References

[1] WHO. Working to overcome the global impact of neglected tropical diseases—Summary. Wkly Epidemiol Rec. 2011;86(13):113–20. Epub 2011/03/29. 21438440

[2] Andrade DV, Gollob KJ, Dutra WO. Acute Chagas Disease: New Global Challenges for an Old Neglected Disease. PLoS Negl Trop Dis. 2014;8(7):e3010 Epub 2014/08/01. PNTD-D-14-00148 [pii]. PubMed Central PMCID: PMC4117453. doi: 10.1371/journal.pntd.0003010 25077613 10.1371/journal.pntd.0003010 PMC4117453

[3] Coura JR, Dias JC. Epidemiology, control and surveillance of Chagas disease: 100 years after its discovery. Mem Inst Oswaldo Cruz. 2009;104 Suppl 1:31–40. Epub 2009/09/24. 19753455 10.1590/s0074-02762009000900006

[4] PAHO. Supply of blood for transfusion in Latin American and Caribbean countries 2012 and 2013. In: HSS/MT, editor. Washington, DC: PAHO; 2015.

[5] Sabino EC, Goncalez TT, Salles NA, Silva GR, Chamone DF. Trends in the prevalence of Chagas' disease among first-time blood donors in Sao Paulo, Brazil. Transfusion. 2003;43(7):853–6. Epub 2003/06/26. 12823743 10.1046/j.1537-2995.2003.t01-2-00432.x

[6] Sabino EC, Salles NA, Sarr M, Barreto AM, Oikawa M, Oliveira CD, et al Enhanced classification of Chagas serologic results and epidemiologic characteristics of seropositive donors at three large blood centers in Brazil. Transfusion. 2010;50(12):2628–37. Epub 2010/06/26. PubMed Central PMCID: PMC2997114. doi: 10.1111/j.1537-2995.2010.02756.x 20576017 10.1111/j.1537-2995.2010.02756.x PMC2997114

[7] Ribeiro AL, Rocha MO. [Indeterminate form of Chagas disease: considerations about diagnosis and prognosis]. Rev Soc Bras Med Trop. 1998;31(3):301–14. Epub 1998/06/05. 9612022 10.1590/s0037-86821998000300008

[8] Rassi A Jr., Rassi A, Marin-Neto JA. Chagas disease. Lancet. 2010;375(9723):1388–402. Epub 2010/04/20. doi: 10.1016/S0140-6736(10)60061-X 20399979 10.1016/S0140-6736(10)60061-X

[9] Sabino EC, Ribeiro AL, Salemi VM, Di Lorenzo Oliveira C, Antunes AP, Menezes MM, et al Ten-year incidence of Chagas cardiomyopathy among asymptomatic Trypanosoma cruzi-seropositive former blood donors. Circulation. 2013;127(10):1105–15. Epub 2013/02/09. PubMed Central PMCID: PMC3643805. doi: 10.1161/CIRCULATIONAHA.112.123612 23393012 10.1161/CIRCULATIONAHA.112.123612 PMC3643805

[10] Barros Pereira C Junior, Markman Filho B. Clinical and echocardiographic predictors of mortality in chagasic cardiomyopathy—systematic review. Arq Bras Cardiol. 2014;102(6):602–10. Epub 2014/07/09. PubMed Central PMCID: PMC4079025. doi: 10.5935/abc.20140068 25004422 10.5935/abc.20140068 PMC4079025

[11] Martins-Melo F, Alencar CH, Ramos AN Jr, Heukelbach J. Epidemiology of Mortality Related to Chaga's Disease in Brazil, 1997–2007. PloS Negl Trop Dis. 2012;6(2):e1508 doi: 10.1371/journal.pntd.0001508 22348163 10.1371/journal.pntd.0001508 PMC3279342

[12] Martins-Melo FR, Ramos Junior AN, Alencar CH, Heukelbach J. Multiple causes of death related to Chagas' disease in Brazil, 1999 to 2007. Rev Soc Bras Med Trop. 2012;45(5):591–6. Epub 2012/11/16. 23152342 10.1590/s0037-86822012000500010

[13] Cucunuba ZM, Okuwoga O, Basanez MG, Nouvellet P. Increased mortality attributed to Chagas disease: a systematic review and meta-analysis. Parasit Vectors. 2016;9(1):42. Epub 2016/01/28. PubMed Central PMCID: PMC4728795. 26813568 10.1186/s13071-016-1315-x PMC4728795

[14] Carneiro-Proietti AB, Sabino EC, Sampaio D, Proietti FA, Goncalez TT, Oliveira CD, et al Demographic profile of blood donors at three major Brazilian blood centers: results from the International REDS-II study, 2007 to 2008. Transfusion. 2010;50(4):918–25. Epub 2009/12/17. PubMed Central PMCID: PMC3079513. doi: 10.1111/j.1537-2995.2009.02529.x 20003051 10.1111/j.1537-2995.2009.02529.x PMC3079513

[15] Salles NA. Risk of exposure to Chagas’ disease among seroreactive Brazilian blood donors Transfusion. 1996;36(11/12):969–73. 8937406 10.1046/j.1537-2995.1996.36111297091740.x

[16] Camargo KR Jr., Coeli CM. [Reclink: an application for database linkage implementing the probabilistic record linkage method]. Cad Saude Publica. 2000;16(2):439–47. Epub 2000/07/07. 10883042 10.1590/s0102-311x2000000200014

[17] Capuani L, Bierrenbach AL, Abreu F, Takecian PL, Ferreira JE, Sabino ES. Accuracy of a probabilistic record-linkage methodology used to track blood donors in the Mortality Information System database. Cad Saúde Pública. 2014;30(8):1623–32. 25210903 10.1590/0102-311x00024914 PMC4863984

[18] StataCorp_LP. Stata/IC 13.1 ed2013.

[19] Pereira JB, Willcox HP, Coura JR. [Morbidity in Chagas' disease. III. Longitudinal study of 6 years, in Virgem da Lapa, MG, Brazil]. Mem Inst Oswaldo Cruz. 1985;80(1):63–71. Epub 1985/01/01. 3937014 10.1590/s0074-02761985000100010

[20] Maguire JH, Hoff R, Sherlock I, Guimaraes AC, Sleigh AC, Ramos NB, et al Cardiac morbidity and mortality due to Chagas' disease: prospective electrocardiographic study of a Brazilian community. Circulation. 1987;75(6):1140–5. Epub 1987/06/01. 3552307 10.1161/01.cir.75.6.1140

[21] Mota EA, Guimaraes AC, Santana OO, Sherlock I, Hoff R, Weller TH. A nine year prospective study of Chagas' disease in a defined rural population in northeast Brazil. Am J Trop Med Hyg. 1990;42(5):429–40. Epub 1990/05/01. 2111098 10.4269/ajtmh.1990.42.429

[22] Pimenta J, Valente N, Miranda M. [Long-term follow up of asymptomatic chagasic individuals with intraventricular conduction disturbances, correlating with non-chagasic patients]. Rev Soc Bras Med Trop. 1999;32(6):621–31. Epub 2000/07/06. 10881098 10.1590/s0037-86821999000600003

[23] Lima-Costa MF, Peixoto SV, Ribeiro AL. Chagas disease and mortality in old age as an emerging issue: 10 year follow-up of the Bambui population-based cohort study (Brazil). Int J Cardiol. 2010;145(2):362–3. Epub 2010/04/20. doi: 10.1016/j.ijcard.2010.02.036 20399519 10.1016/j.ijcard.2010.02.036

[24] Lima-Costa MF, Matos DL, Ribeiro AL. Chagas disease predicts 10-year stroke mortality in community-dwelling elderly: the Bambui cohort study of aging. Stroke. 2010;41(11):2477–82. Epub 2010/09/25. doi: 10.1161/STROKEAHA.110.588061 20864663 10.1161/STROKEAHA.110.588061

[25] Schmidt MI, Duncan BB, Ishitani L, da Conceicao Franco G, de Abreu DM, Lana GC, et al Trends in mortality due to diabetes in Brazil, 1996–2011. Diabetol Metab Syndr. 2015;7:109 Epub 2015/12/01. PubMed Central PMCID: PMC4661935. doi: 10.1186/s13098-015-0105-5 26617678 10.1186/s13098-015-0105-5 PMC4661935

[26] Franca E, de Abreu DX, Rao C, Lopez AD. Evaluation of cause-of-death statistics for Brazil, 2002–2004. Int J Epidemiol. 2008;37(4):891–901. Epub 2008/07/26. doi: 10.1093/ije/dyn121 18653516 10.1093/ije/dyn121

[27] Jorge MH, Laurenti R, Gotlieb SL. [Quality analysis of Brazilian vital statistics: the experience of implementing the SIM and SINASC systems]. Cien Saude Colet. 2007;12(3):643–54. Epub 2007/08/08. 17680121 10.1590/s1413-81232007000300014

[28] Jorge MH, Laurenti R, Di Nubila HB. [Death and its epidemiological investigation: considerations about some relevant aspects]. Rev Bras Epidemiol. 2010;13(4):561–76. Epub 2010/12/25. 21180846 10.1590/s1415-790x2010000400002

[29] Goncalez T, Sabino EC, Charmone DF. Trends in the profile of blood donors at a large blood center in the city of São Paulo, Brazil. Rev Panam Salud Publica. 2003;13(2–3):144–8. 12744790 10.1590/s1020-49892003000200016

[30] Baeninger R. São Paulo e suas migrações no final do século 20. Sao Paulo em Perspectiva. 2005;19(3):84–96.

[31] Acquatella H, Catalioti F, Gomez-Mancebo JR, Davalos V, Villalobos L. Long-term control of Chagas disease in Venezuela: effects on serologic findings, electrocardiographic abnormalities, and clinical outcome. Circulation. 1987;76(3):556–62. Epub 1987/09/01. 2957109 10.1161/01.cir.76.3.556

[32] Ianni BM, Arteaga E, Frimm CC, Pereira Barretto AC, Mady C. Chagas' heart disease: evolutive evaluation of electrocardiographic and echocardiographic parameters in patients with the indeterminate form. Arq Bras Cardiol. 2001;77(1):59–62. Epub 2001/08/14. 11500748 10.1590/s0066-782x2001000700006

[33] Mady C, Ianni BM, de Souza JL Jr. Benznidazole and Chagas disease: can an old drug be the answer to an old problem? Expert Opin Investig Drugs. 2008;17(10):1427–33. Epub 2008/09/24. doi: 10.1517/13543784.17.10.1427 18808305 10.1517/13543784.17.10.1427

