# Correction: Mortality among blood donors seropositive and seronegative for Chagas disease (1996–2000) in São Paulo, Brazil: A death certificate linkage study

**DOI:** 10.1371/journal.pntd.0008871

**Published:** 2020-11-06

**Authors:** Ligia Capuani, Ana Luiza Bierrenbach, Airlane Pereira Alencar, Alfredo Mendrone, João Eduardo Ferreira, Brian Custer, Antonio Luiz P. Ribeiro, Ester Cerdeira Sabino

There is an error in the article XML causing the third author’s initials to be indexed incorrectly. The correct initials are: Alencar AP. The correct citation is: Capuani L, Bierrenbach AL, Alencar AP, Mendrone A Jr, Ferreira JE, Custer B, et al. (2017) Mortality among blood donors seropositive and seronegative for Chagas disease (1996–2000) in São Paulo, Brazil: A death certificate linkage study. PLoS Negl Trop Dis 11(5): e0005542. https://doi.org/10.1371/journal.pntd.0005542

There is an error in the article XML causing the seventh author’s initials to be indexed incorrectly. The correct initials are: Ribeiro ALP. There is an error in the article XML causing the eighth author’s initials to be indexed incorrectly. The correct initials are: Sabino EC.
